# Supplementary figures and images for: Heterologous prime-boost immunization based on a human adenovirus 5 vectored containing Trichinella spiralis Cystatin-like protein elicits protective mucosal immunity in mice
Source: PLoS Negl Trop Dis. 2025 Jul 16;19(7):e0013323. doi: 10.1371/journal.pntd.0013323 (PMC12279143; doi:10.1371/journal.pntd.0013323)

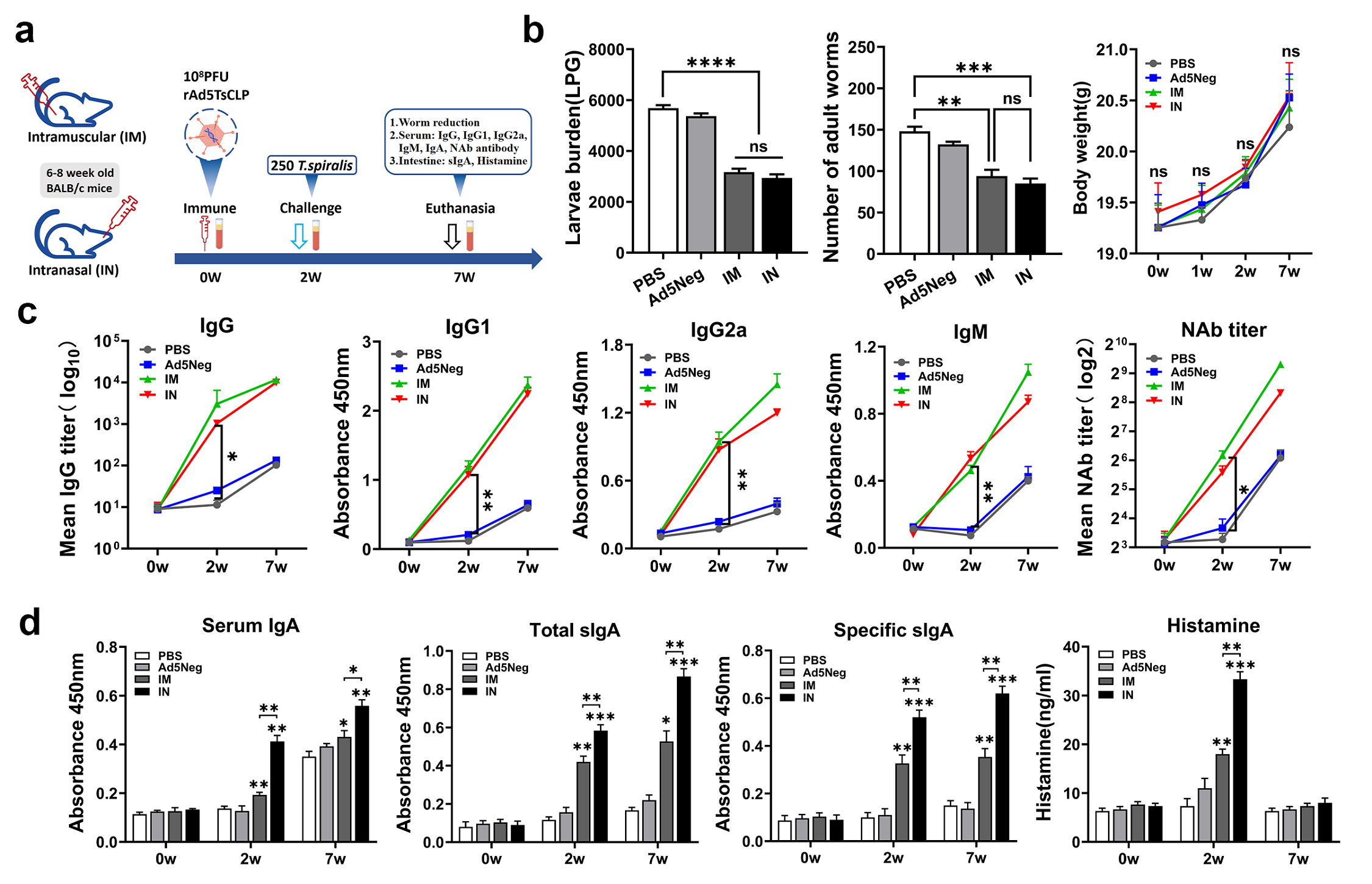

Supplement: S1 Fig — (a). A scheme of experiments. (b) Protective analyses of rAd5TsCLP immunization in mice challenged with 250 Trichinella spiralis muscle larvae by measuring larval burden (LPG), adult worm counts (Ad3), and changes in body weight. (c) Serum levels of IgG, IgG1, IgG2a, IgA, IgM, and NAb antibodies in mice. (d) IgA antibody level in serum, sIgA antibody and histamine level in intestinal lavage fluid of mice. P values were analyzed by one-way ANOVA for statistical differences (n = 10) (* P < 0.05; ** P < 0.01; *** P < 0.001, **** P < 0.0001). (TIF) [file pntd.0013323.s001.tif]

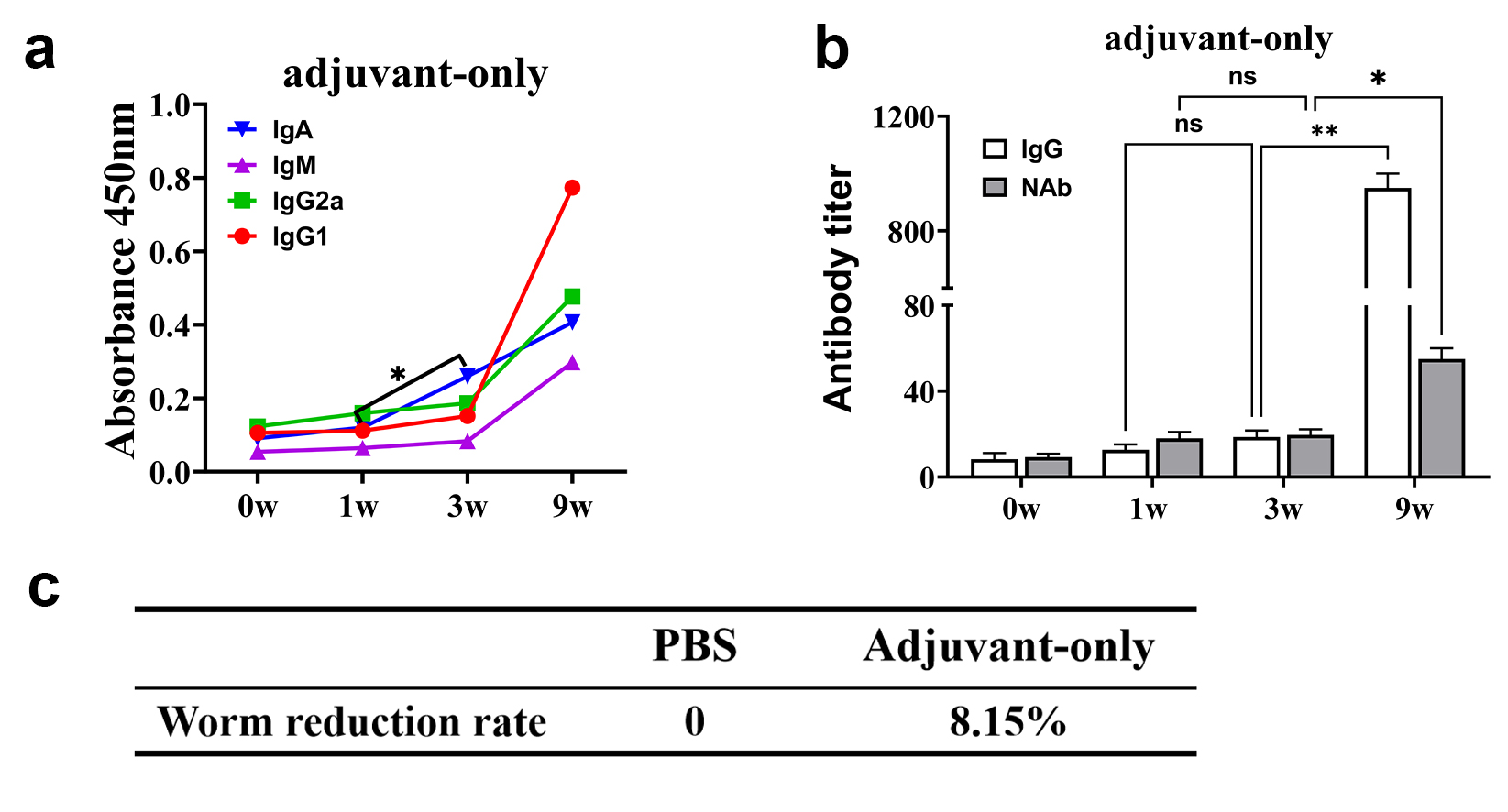

Supplement: S2 Fig — (a) Serum levels of IgG1, IgG2a, IgA and IgM antibodies in mice. (b) Serum levels of total IgG and neutralizing antibody (NAb) titers in mice.(c) Protective efficacy of the adjuvant-only group compared to the PBS control group, evaluated by worm reduction rate. P values were analyzed by two-way ANOVA for statistical differences (n = 10) (* P < 0.05; ** P < 0.01). (TIF) [file pntd.0013323.s002.tif]

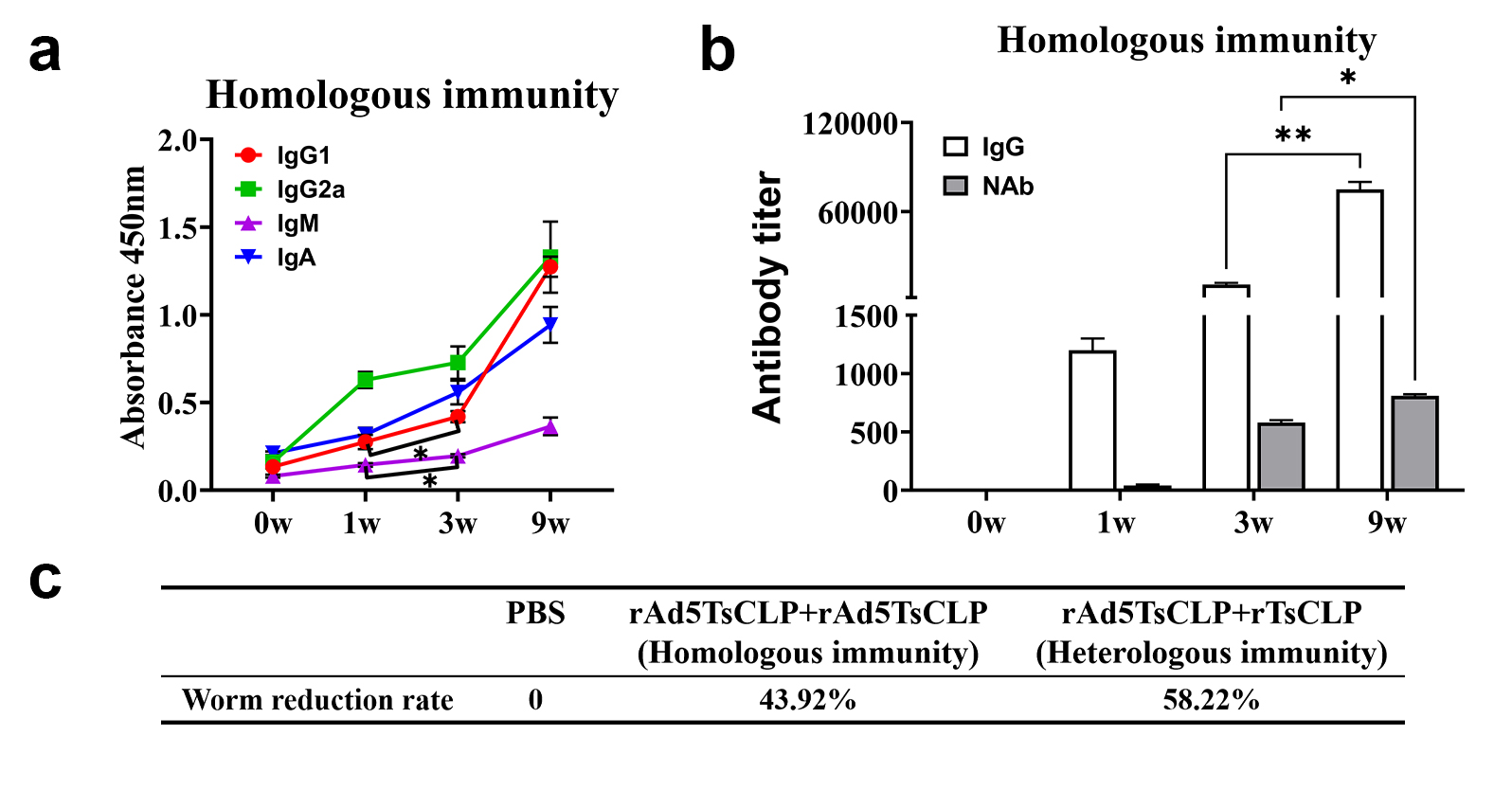

Supplement: S3 Fig — (a) Serum levels of IgG1, IgG2a, IgA and IgM antibodies in mice. (b) Serum levels of total IgG and neutralizing antibody (NAb) titers in mice.(c) Protective efficacy of the homologous immunization group compared to the PBS control group, evaluated by worm reduction rate. P values were analyzed by two-way ANOVA for statistical differences (n = 10) (* P < 0.05; ** P < 0.01; *** P < 0.001, **** P < 0.0001). (TIF) [file pntd.0013323.s003.tif]
