# Supplementary material for: Heterologous prime-boost immunization based on a human adenovirus 5 vectored containing Trichinella spiralis Cystatin-like protein elicits protective mucosal immunity in mice
Source: PLoS Negl Trop Dis. 2025 Jul 16;19(7):e0013323. doi: 10.1371/journal.pntd.0013323 (PMC12279143; doi:10.1371/journal.pntd.0013323)
Supplement: Attachment — Submitted filename: 2025-5-6-Comments on PNTD-D-25-00587PNTD.docx [file pntd.0013323.s006.docx]

**Reviewer comments on PNTD-D-25-00587**

In this manuscript titled “Heterologous prime-boost immunization based on a human adenovirus 5 vectored containing *Trichinella spiralis* Cystatin-like protein elicits protective mucosal immunity in mice”(PNTD-D-25-00587), the authors dressed an important problem. The objectives of this study are clear and the experimental design is appropriate. Overall, I recommend that this manuscript could be accepted for publication after some minor revisions. They are as follows:

1. General comment: Ensure consistent use of *italics* for species or genus names (e.g., *Trichinella spiralis,*); Include in your description if you talk about larvae or adult worms (in the discussion thread as well). And the Latin names of the genus in the references, Salmonella typhimurium-> Salmonella typhimurium in italics.
2. Line 55: put a space before the square brackets [], such as larvae[1]-> larvae [1].
3. Line76. Provide a brief description of the basic structural features of adenovirus (e.g., capsid organization, genome structure) to contextualize the vector design for readers unfamiliar with adenoviral systems.
4. Line92-94. Previous published studies should be cited to support that cystatin-like proteins from *Trichinella spiralis* are localized in β-stichocytes and expressed in various developmental stages, including the early invasion and muscle larval stages (Tang et al., 2015; Guiliano et al., 2009).
a) Tang B, Liu M, Wang L, Yu S, Shi H, Boireau P, Cozma V, Wu X, Liu X. Characterisation of a high-frequency gene encoding a strongly antigenic cystatin-like protein from *Trichinella spiralis* at its early invasion stage. Parasit Vectors. 2015; 8:78. doi: 10.1186/s13071-015-0689-5.
b) Guiliano DB, Oksov Y, Lustigman S, Gounaris K, Selkirk ME. Characterisation of novel protein families secreted by muscle stage larvae of *Trichinella spiralis*. Int J Parasitol. 2009;39(5):515-24. doi: 10.1016/j.ijpara.2008.09.012. Epub 2008 Oct 21.
5. Line182. Please verify the selection of adjuvant CpG1018 over other adjuvants (e.g., its mechanism of action as a TLR9 agonist and relevance to mucosal immunity).
6. The authors should explicitly specify whether the control group received the same 250 muscle larvae as the infection group to ensure consistency in experimental design and rule out volume-dependent effects.
7. Line398. While cytokine profiling is comprehensive, the roles of IL-12p70, MCP-1, and IL-17 in the observed immune response should be explicitly presented in the results section.
8. Figure 6c. Correct the x-axis label "Ad*Ts*CLP:r*Ts*CLP" as "rAd5*Ts*CLP:r*Ts*CLP" to align with the nomenclature used in the Methods section.
9. Suggest the authors to clarify whether the term 'Special sIgA' in Figure 4 refers to '*Ts*CLP-specific sIgA' and revise the figure accordingly for consistency with the legend, and to clearly state the statistical methods used in Figure 3, including the type of test and significance thresholds in the figure legend.
