# Supplementary material for: Heterologous prime-boost immunization based on a human adenovirus 5 vectored containing Trichinella spiralis Cystatin-like protein elicits protective mucosal immunity in mice
Source: PLoS Negl Trop Dis. 2025 Jul 16;19(7):e0013323. doi: 10.1371/journal.pntd.0013323 (PMC12279143; doi:10.1371/journal.pntd.0013323)
Supplement: Attachment — Submitted filename: Questions.docx [file pntd.0013323.s007.docx]

Line 119. This paragraph is somewhat unclear, as it states that parasite maintenance is carried out in Wistar rats, while larval collection is performed in mice. Could you clarify the methodology?

Line 179. Was the inclusion of an adjuvant-only control group considered in the experimental design?

Line 187. As I understand it, the booster immunizations were consistently administered intramuscularly with rTsCLP; was revaccination with rAd5TsCLP via the intranasal route considered at any point?

Line 234. It should be noted that the mice were previously anesthetized before blood collection from the orbital venous plexus."

Line 249. Was the goblet cell count performed using Hematoxylin and Eosin staining, or was another more specific stain, such as Alcian blue, used?

Line 254. It would be appropriate to specify the methodology used for the recovery of adult worms from the intestine, as well as the procedure for obtaining muscle larvae.

Line 377. Some studies suggest that Trichinella spiralis infection naturally induces an increase in goblet cell numbers; however, due to endoplasmic reticulum stress, these cells may be unable to adequately produce mucins, thereby compromising their protective role. In your study, I would recommend assessing mucin expression, as an increase in goblet cell numbers alone is not necessarily indicative of enhanced protection..

Line 410. Why were cervical lymph nodes analyzed instead of mesenteric lymph nodes?
