# Supplementary material for: Heterologous prime-boost immunization based on a human adenovirus 5 vectored containing Trichinella spiralis Cystatin-like protein elicits protective mucosal immunity in mice
Source: PLoS Negl Trop Dis. 2025 Jul 16;19(7):e0013323. doi: 10.1371/journal.pntd.0013323 (PMC12279143; doi:10.1371/journal.pntd.0013323)
Supplement: Attachment — Submitted filename: Response to Reviewers.docx [file pntd.0013323.s009.docx]

Dear editor,

Thank you for giving us a chance to revise and improve our manuscript. On behalf of all co-authors, I would like to extend our heartfelt thanks to you and the reviewers for the favorable and constructive comments on our manuscript titled “**Heterologous prime-boost immunization based on a human adenovirus 5 vectored containing *Trichinella spiralis* Cystatin-like protein elicits protective mucosal immunity in mice**”(**Manuscript Number: PNTD-D-25-00587**). We have revised and improved the manuscript strictly according to the reviewer’s comments. We are confident that the paper is greatly improved and hope this manuscript will be finally acceptable for publication in “***PLOS Neglected Tropical Diseases***”*.*

The following is a point-by-point response to the referees' comments and indicates where the modifications have been made. All changes in the revised manuscript were highlighted with red color.

Regards and best wishes,

Dr. Xue Bai

State Key Laboratory for Diagnosis and Treatment of Severe Zoonotic Infectious Diseases, Key Laboratory for Zoonosis Research of the Ministry of Education, Institute of Zoonosis, College of Veterinary Medicine, Jilin University, Changchun 130062, China

E-mail: [namiya23@163.com](mailto:namiya23@163.com), baixue2008851001@jlu.edu.cn

**Responses to Journal Requirements:**

**Question 1:** Please upload all main figures as separate Figure files in .tif or .eps format. For more information about how to convert and format your figure files please see our guidelines: <https://journals.plos.org/plosntds/s/figures>

Response 1: Thank you for this suggestion. We have converted all main figures to high-resolution .TIFF files and uploaded them as separate figure files in accordance with the guidelines.

**Question 2:** We have noticed that you have uploaded Supporting Information files, but you have not included a list of legends. Please add a full list of legends for your Supporting Information files after the references list.

Response 2: Thank you for pointing this out. We have added the “List of Legends for Supporting Information” immediately after the References section, and uploaded the revised Supporting Information file.

**Question 3:** Some material included in your submission may be copyrighted. According to PLOSu2019s copyright policy, authors who use figures or other material (e.g., graphics, clipart, maps) from another author or copyright holder must demonstrate or obtain permission to publish this material under the Creative Commons Attribution 4.0 International (CC BY 4.0) License used by PLOS journals. Please closely review the details of PLOSu2019s copyright requirements here: PLOS Licenses and Copyright. If you need to request permissions from a copyright holder, you may use PLOS's Copyright Content Permission form.

Please respond directly to this email and provide any known details concerning your material's license terms and permissions required for reuse, even if you have not yet obtained copyright permissions or are unsure of your material's copyright compatibility. Once you have responded and addressed all other outstanding technical requirements, you may resubmit your manuscript within Editorial Manager.

Potential Copyright Issues:

- Figures 3 and S1. Please confirm whether you drew the images / clip-art within the figure panels by hand. If you did not draw the images, please provide (a) a link to the source of the images or icons and their license / terms of use; or (b) written permission from the copyright holder to publish the images or icons under our CC BY 4.0 license. Alternatively, you may replace the images with open source alternatives. See these open source resources you may use to replace images / clip-art:

- https://commons.wikimedia.org

- https://openclipart.org/.

Response 3: Thank you for raising this issue. Figures 3 and S1 were independently designed and generated by the authors, no copyright issues are involved.

**Question 4:** In the online submission form, you indicated that "All data are available from the corresponding author upon request.". All PLOS journals now require all data underlying the findings described in their manuscript to be freely available to other researchers, either

- In a public repository

- Within the manuscript itself

- Uploaded as supplementary information.

This policy applies to all data except where public deposition would breach compliance with the protocol approved by your research ethics board. If your data cannot be made publicly available for ethical or legal reasons (e.g., public availability would compromise patient privacy), please explain your reasons by return email and your exemption request will be escalated to the editor for approval. Your exemption request will be handled independently and will not hold up the peer review process, but will need to be resolved should your manuscript be accepted for publication. One of the Editorial team will then be in touch if there are any issues.

Response 4: Thank you for the reminder. All raw and processed data have been deposited in Figshare (DOI: 10.6084/m9.figshare.28822889).

We revised the Data availability statement section as follows: “ All data are fully available within the manuscript, Supporting Information, and Figshare (DOI: 10.6084/m9.figshare.28822889) (not released public).” in the revised manuscript (**Lines 669-670**).

**Question 5:** Please ensure that the funders and grant numbers match between the Financial Disclosure field and the Funding Information tab in your submission form. Note that the funders must be provided in the same order in both places as well.

- State the initials, alongside each funding source, of each author to receive each grant. For example: "This work was supported by the National Institutes of Health (####### to AM; ###### to CJ) and the National Science Foundation (###### to AM)."

- State what role the funders took in the study. If the funders had no role in your study, please state: "The funders had no role in study design, data collection and analysis, decision to publish, or preparation of the manuscript.".

If you did not receive any funding for this study, please simply state: u201cThe authors received no specific funding for this work.u201d

Response 5: Thank you for your advice. We have carefully checked the Financial Disclosure field and the Funding Information. We revised the Funding section as follows: “XB prof. was supported by National Key Research and Development Program of China (2023YFE0107300); BT prof. was supported by National Key Research and Development Program of China (2023YFD1802400); ML prof. was supported by the National Natural Science Foundation of China (NSFC32230104, 32373032 and 82201959), XL prof. was supported by the Science and Technology Talents and Platform Plan of Yunnan Province (Academician and Expert Workstation, 202305AF150167). The funders had no role instudy design, data collection and analysis, decision to publish, or preparation of the manuscript.” in the revised manuscript (**Lines 693-700**).

**Responses to Reviewer #1:**

**Question 1:** General comment: Ensure consistent use of italics for species or genus names (e.g., *Trichinella spiralis*,); Include in your description if you talk about larvae or adult worms (in the discussion thread as well). And the Latin names of the genus in the references, Salmonella typhimurium-> Salmonella typhimurium in italics.

Response 1: Thank you for your advice. We have consistently italicized all genus and species names (including *Trichinella spiralis*, *Salmonella typhimurium*, etc.), and distinguished common terms such as "adult worms" and "muscle larvae." Additionally, all Latin names in the References section have also been italicized in our revised manuscript.

**Question 2:** Line 55: put a space before the square brackets [], such as larvae[1]-> larvae [1].

Response 2: Thank you for your advice. We have added a space before the square brackets in the revised manuscript.

**Question 3:** Line76. Provide a brief description of the basic structural features of adenovirus (e.g., capsid organization, genome structure) to contextualize the vector design for readers unfamiliar with adenoviral systems.

Response 3: Thank you for your advice. Under your suggestion, we have added a concise description of adenoviral structural features in the background as follows: “Adenovirus is a non-encapsulated spherical structured virus with a diameter of about 70-90 nm, consisting of 252 capsid particles arranged in an icosahedral arrangement and containing linear double-stranded DNA molecules inside [Gallardo et al., 2021; Li et al., 2025 ].”in the revised manuscript (**Lines 76-78**).

**References:**

[1] **Gallardo J, Pérez-Illana M, Martín-González N, San Martín C. Adenovirus Structure: What Is New? Int J Mol Sci. 2021 May 15;22(10):5240. doi: 10.3390/ijms22105240. PMID: 34063479; PMCID: PMC8156859.**
[2] **Li H, Shao L, Liu Z, Liu Q, Xiang Y. Molecular mechanisms of the viral encoded chaperone 100K in capsid folding and assembly of adenovirus. Nat Commun. 2025 Apr 29;16(1):4013. doi: 10.1038/s41467-025-59301-4. PMID: 40301360; PMCID: PMC12041577.**

**Question 4:** Line92-94. Previous published studies should be cited to support that cystatin-like proteins from *Trichinella spiralis* are localized in β-stichocytes and expressed in various developmental stages, including the early invasion and muscle larval stages (Tang et al., 2015; Guiliano et al., 2009).

a)Tang B, Liu M, Wang L, Yu S, Shi H, Boireau P, Cozma V, Wu X, Liu X. Characterisation of a high-frequency gene encoding a strongly antigenic cystatin-like protein from *Trichinella spiralis* at its early invasion stage. Parasit Vectors. 2015; 8:78. doi: 10.1186/s13071-015-0689-5.

b)Guiliano DB, Oksov Y, Lustigman S, Gounaris K, Selkirk ME. Characterisation of novel protein families secreted by muscle stage larvae of *Trichinella spiralis*. Int J Parasitol. 2009;39(5):515-24. doi: 10.1016/j.ijpara.2008.09.012. Epub 2008 Oct 21.

Response 4: Thank you for your suggestion. We have cited the articles in our revised manuscript (**Line 95**).

**Question 5:** Line182. Please verify the selection of adjuvant CpG 1018 over other adjuvants (e.g., its mechanism of action as a TLR9 agonist and relevance to mucosal immunity)

Response 5: Thank you for your suggestion. We have provided a more explicit and detailed explanation as follows: “Toll-like receptor (TLR) agonist adjuvants have demonstrated significant advantages when used with adenoviral vectors [Zhao et al., 2023]. Therefore, we selected the TLR9 agonist CpG 1018 as our adjuvant to enhance the efficacy of the r*Ts*CLP vaccine by boosting cellular immune responses [Facciolà et al., 2022]. CpG 1018 induces Th1-biased systemic and mucosal immunity through the MyD88–IRF7/NF-κB signaling pathways. It has demonstrated clinical efficacy and safety as the active component of the licensed HEPLISAV-B hepatitis B vaccine, which also offers antigen dose reduction advantages [McKoy et al., 2025]. Furthermore, CpG 1018 exhibits superior immunostimulatory activity compared to traditional adjuvants in both systemic and mucosal immune compartments.” in the revised manuscript (**Lines 641-650**).

**References:**

[1] **Zhao T, Cai Y, Jiang Y, He X, Wei Y, Yu Y, Tian X. Vaccine adjuvants: mechanisms and platforms. Signal Transduct Target Ther. 2023 Jul 19;8(1):283. doi: 10.1038/s41392-023-01557-7. PMID: 37468460; PMCID: PMC10356842.**
[2] **Facciolà A, Visalli G, Laganà A, Di Pietro A. An Overview of Vaccine Adjuvants: Current Evidence and Future Perspectives. Vaccines (Basel). 2022 May 22;10(5):819. doi: 10.3390/vaccines10050819. PMID: 35632575; PMCID: PMC9147349.**
[3] **McKoy K, Campbell S, Novy P, Janssen RS. Hepatitis B vaccination with HepB-CpG in people living with HIV: a narrative review. Expert Rev Vaccines. 2025 Dec;24(1):365-372. doi: 10.1080/14760584.2025.2502643. Epub 2025 May 12. PMID: 40336183.**

**Question 6:** The authors should explicitly specify whether the control group received the same 250 muscle larvae as the infection group to ensure consistency in experimental design and rule out volume-dependent effects.

Response 6: Thank you for your advice. In our methodology, both infection and control groups received identical intragastric inoculations of 250 muscle larvae, which were counted manually.

**Question 7:** Line398. While cytokine profiling is comprehensive, the roles of IL-12p70, MCP-1, and IL-17 in the observed immune response should be explicitly presented in the results section.

Response 7: Thank you for your insightful comment. We have added a detailed explanation of the roles of IL-12p70, MCP-1, and IL-17 in the Results section as follows: “Notably, GM-CSF increased 11-fold, suggesting enhanced activation and differentiation of myeloid-derived antigen-presenting cells. IL-12p70, a pivotal cytokine for promoting Th1 differentiation and stimulating IFN-γ production by T and NK cells, was elevated 5-fold, underscoring its central role in initiating Th1-skewed cellular immunity. In parallel, elevated MCP-1 and IL-17 indicated enhanced monocyte recruitment and a moderate involvement of the Th17 axis.” in the revised manuscript (**Lines 432-438**).

**Question 8:** Figure 6c. Correct the x-axis label "Ad*Ts*CLP:r*Ts*CLP" as "rAd5*Ts*CLP:r*Ts*CLP" to align with the nomenclature used in the Methods section.

Response 8: Thank you for your advice. We have corrected the x-axis label in Figure 6c by changing “Ad*Ts*CLP:r*Ts*CLP” to “rAd5*Ts*CLP:r*Ts*CLP”, the updated Figure 6c is shown below.

Fig 6. Prime-boost immunization enhances T lymphocyte activation in the spleen.

**Question 9:** Suggest the authors to clarify whether the term 'Special sIgA' in Figure 4 refers to '*Ts*CLP-specific sIgA' and revise the figure accordingly for consistency with the legend, and to clearly state the statistical methods used in Figure 3, including the type of test and significance thresholds in the figure legend.

Response 9: Thank you for this valuable suggestion.

(1) In our experiments, the term 'Special sIgA' specifically refers to *Ts*CLP-specific sIgA." For consistency, we modified Figure 4a as shown below.

Fig 4. Enhanced mucosal immune responses and increased goblet cell density following rAd5*Ts*CLP:r*Ts*CLP immunization.

(2) We added the statistical methods to the legend of Figure 3 as follows: “ Data are presented as mean ± SEM (n = 10). Statistical analysis was performed using two-way ANOVA (* *P* < 0.05; ** *P* < 0.01; *** *P* < 0.001, **** *P* < 0.0001).” in the revised manuscript (**Line 381-382**).

**Responses to Reviewer #2:**

**Question 1:** Line 119. This paragraph is somewhat unclear, as it states that parasite maintenance is carried out in Wistar rats, while larval collection is performed in mice. Could you clarify the methodology?

Response 1: Thank you for your comment. In the *Trichinella* experimental protocol, both rats and mice serve as suitable hosts for *T. spiralis* propagation and strain maintenance. Improving the recovery rate of *Trichinella spiralis* muscle larvae, we established pre-infections in rats to collect over 10000 muscle larvae for subsequent mouse challenge. We have revised the “Parasites and Cells” of the Methods section as follows: “ The ISS534 strain of *T. spiralis* was maintained in female Wistar rats by oral administration of 3000 larvae [28], and subsequently used for challenge infections in mice. Muscle larvae (ML) were harvested 35 days post-infection from infected mice muscle tissue *via* the pepsin-HCl digestion method [29].” in the revised manuscript (**Lines 123-124**).

**Question 2:** Line 179. Was the inclusion of an adjuvant-only control group considered in the experimental design?

Response 2: Thank you for your suggestion. During the initial experimental design, we included a CpG 1018 adjuvant-only group. However, this group did not elicit substantial immunogenicity or protective efficacy. To enhance the clarity of the experimental results, the results from all five groups are presented in the manuscript, referencing relevant literature [Perera et al., 2022].

Your advice is highly valuable, we have included these experimental results in the Supporting Information. The antibody levels and protective efficacy in the adjuvant-only group, including the worm reduction rate relative to the PBS group, are summarized as follows (Fig. S2):

**References:**

[1] **Perera DJ, Hassan AS, Liu SS, Elahi SM, Gadoury C, Weeratna RD, Gilbert R, Ndao M. A low dose adenovirus vectored vaccine expressing Schistosoma mansoni Cathepsin B protects from intestinal schistosomiasis in mice. EBioMedicine. 2022 Jun;80:104036. doi: 10.1016/j.ebiom.2022.104036.**

**Question 3:** Line 187. As I understand it, the booster immunizations were consistently administered intramuscularly with r*Ts*CLP; was revaccination with rAd5*Ts*CLP *via* the intranasal route considered at any point?

Response 3: Thank you for your insightful question. Our initial experimental design included a two-dose adenovirus group. Compared to the prime-boost immunization strategy, the results of homologous group exhibited lower antibody levels and worm reduction rate (as shown below). Meanwhile, current studies have reported that repeated administration of adenoviral vectors may induce pre-existing immunity, which can compromise vaccine efficacy [Zaiss et al., 2009; Wang et al., 2022]. Therefore, these experimental results of the homologous group were not included in the main text.

Your advice is highly valuable, we have included these experimental results in the Supporting Information. The antibody levels and protective efficacy in the homologous immunity group, including the worm reduction rate compared to the PBS group, the results as follows (Fig. S3):

**References:**

[1] **Zaiss AK, Machado HB, Herschman HR. The influence of innate and pre-existing immunity on adenovirus therapy. J Cell Biochem. 2009 Nov 1;108(4):778-90. doi: 10.1002/jcb.22328.**
[2] **Wang WC, Sayedahmed EE, Mittal SK. Significance of Preexisting Vector Immunity and Activation of Innate Responses for Adenoviral Vector-Based Therapy. Viruses. 2022 Dec 6;14(12):2727. doi: 10.3390/v14122727.**

**Question 4:** Line 234. It should be noted that the mice were previously anesthetized before blood collection from the orbital venous plexus."

Response 4: Thank you for your careful review. We anesthetized the mice prior to blood collection from the orbital venous plexus and have revised the “Flow cytometry” of the Methods section as follows: “Mice were anesthetized with 2% sodium pentobarbital (40-50 mg/kg), and adequate anesthesia was confirmed. Blood was collected from the orbital venous plexus, and euthanasia was performed by cervical dislocation under deep anesthesia.” in the revised manuscript (**Lines 243-245**).

**Question 5:** Line 249. Was the goblet cell count performed using Hematoxylin and Eosin staining, or was another more specific stain, such as Alcian blue, used?

Response 5: Thank you for your advice. Under your suggestion, in order to evaluate mucin secretion and goblet cell abundance, we performed an Alcian Blue—Periodic Acid-Schiff (AB-PAS) staining, replacing the HE staining. The revised section of “Methods, Results, Figure legend” as follow:

(1) “Small Intestine and Muscle Histopathology” of the Methods as follows: “After euthanasia, the diaphragm and small intestine were isolated from the mice, dissected free of surface fascia, and rinsed with ice-cold PBS. The tissues were then fixed in 4% paraformaldehyde (PFA) for 24 hours, embedded in paraffin, and sectioned. Sections were stained with hematoxylin and eosin (H&E), as well as with an Alcian Blue Periodic acid Schiff (AB-PAS) kit (Solarbio, China), and images were captured using a bright-field microscope (Olympus, Japan). For the intestinal samples, goblet cells were counted under a multiple high-power field microscope (Olympus, Japan).” in the revised manuscript (**Line 259-260**).

(2) “Intestinal mucosal immune response elicited by rAd5*Ts*CLP:r*Ts*CLP vaccination” of the Results as follows: “Goblet cells are essential components of the intestinal epithelium, responsible for synthesizing and secreting mucins that contribute to the formation of a protective mucus barrier. In this study, AB-PAS staining was performed to simultaneously visualize goblet cells and mucin production in intestinal sections. Compared with the control groups, the rAd5*Ts*CLP:r*Ts*CLP group exhibited a substantial increase in goblet cell density, along with prominent blue-stained acidic mucin accumulation within the intestinal mucosa (Fig. 4b). In contrast, other immunized groups such as r*Ts*CLP:r*Ts*CLP and Ad5Neg:r*Ts*CLP showed only moderate goblet cell expansion and relatively limited mucin secretion (*P* < 0.001). These observations suggest that the rAd5*Ts*CLP:r*Ts*CLP sequential immunization strategy not only enhances goblet cell proliferation but also preserves their secretory functionality, thereby contributing to improved mucosal barrier integrity and potentially augmenting local immune protection against *Trichinella spiralis* infection.” in the revised manuscript (**Lines 396-408**).

(3) The revised Figure 4 legend as follows: “Representative images of small intestinal sections from each group after challenge (AB-PAS staining). Goblet cells are more abundant in the rAd5*Ts*CLP:r*Ts*CLP-vaccinated mice compared to others. Scale bars are 100 μm.” in the revised manuscript (**Lines 417-419**).

Fig 4. Enhanced mucosal immune responses and increased goblet cell density following rAd5*Ts*CLP:r*Ts*CLP immunization.

**Question 6:** Line 254. It would be appropriate to specify the methodology used for the recovery of adult worms from the intestine, as well as the procedure for obtaining muscle larvae.

Response 6: Thank you for your advice. We have provided a detailed description of “the recovery of adult worms from the intestine, as well as the procedure for obtaining muscle larvae,” and have cited the relevant references [Wang et al., 2025; Gajadhar et al., 2019]. We have revised the “Evaluation of worm burden” section of the Methods as follows: “Mice were infected orally with 250 *T. spiralis* muscle larvae (ML) were euthanized at 3 days post-infection for intestinal adult worm (Ad3) and at 5 weeks post-infection for muscle larvae burden (larvae per gram tissue, LPG) determination. For adult worms, small intestines were incubated in 500 mL pre-warmed (37 °C) antibiotic saline for 3 h, allowed to settle 1 h at room temperature, then sedimented Ad3 were washed and counted under a stereomicroscope. For muscle larvae, skeletal muscle was digested in 500 mL of 1% (w/v) pepsin-1% (v/v) HCl at 37 °C with stirring for 2 h, filtered, settled, washed, and ML were counted microscopically.”in the revised manuscript (**Lines 265-272**).

**References:**

[1] **Wang BN, Zhang XZ, Wu JY, Zhang ZY, Cong PK, Zheng WW, Long SR, Liu RD, Cui J, Wang ZQ. Vaccination of mice with *Trichinella spiralis* C-type lectin elicited the protective immunity and enhanced gut epithelial barrier function. PLoS Negl Trop Dis. 2025 Jan 22;19(1):e0012825. doi: 10.1371/journal.pntd.0012825. PMID: 39841790; PMCID: PMC11761079.**
[2] **Gajadhar AA, Noeckler K, Boireau P, Rossi P, Scandrett B, Gamble HR. International Commission on Trichinellosis: Recommendations for quality assurance in digestion testing programs for *Trichinella*. Food Waterborne Parasitol. 2019 Jun 5;16:e00059. doi: 10.1016/j.fawpar.2019.e00059. PMID: 32095629; PMCID: PMC7033998.**

**Question 7:** Line 377. Some studies suggest that *Trichinella spiralis* infection naturally induces an increase in goblet cell numbers; however, due to endoplasmic reticulum stress, these cells may be unable to adequately produce mucins, thereby compromising their protective role. In your study, I would recommend assessing mucin expression, as an increase in goblet cell numbers alone is not necessarily indicative of enhanced protection.

Response 7: Thank you for your suggestion. I agree with your suggestion regarding the“ an increase in goblet cell numbers alone is not necessarily indicative of enhanced protection.” Following your advice, we have added Alcian Blue—Periodic Acid-Schiff (AB-PAS) staining. The results are consistent with our **Response 5**. We have revised the Methods **(Lines 259-260)**, the Results **(Lines 396-408)**, and the Figure legend **(Lines 417-419)**.

**Question 8:** Line 410. Why were cervical lymph nodes analyzed instead of mesenteric lymph nodes?

Response 8: Thank you for this important question. In our study, we used a prime-boost immunization strategy (intranasal priming followed by intramuscular boosting) and evaluated both systemic and mucosal immune responses. Given the intranasal route of administration, we selected the cervical lymph nodes for analysis, as they are the primary draining lymphoid tissues of the upper respiratory tract and directly reflect local antigen-specific T and B cell responses induced by nasal immunization. Although the recombinant adenovirus used in our study is replication-deficient and does not replicate in host cells, it can still effectively deliver the antigen to mucosal sites and initiate robust local immune responses. This approach aligns with established methodologies in mucosal vaccine research [Hsieh et al., 2021; Diallo et al., 2023]. We maintain that this approach suffices to assess mucosal immunity in our vaccine model.

**References:**

[1] **Hsieh MS, Hsu CW, Tu LL, Chai KM, Yu LL, Wu CC, Chen MY, Chiang CY, Liu SJ, Liao CL, Chen HW. Intranasal Vaccination With Recombinant Antigen-FLIPr Fusion Protein Alone Induces Long-Lasting Systemic Antibody Responses and Broad T Cell Responses. Front Immunol. 2021 Oct 11;12:751883. doi: 10.3389/fimmu.2021.751883. PMID: 34707615; PMCID: PMC8543008.**
[2] **Diallo BK, Ní Chasaide C, Wong TY, Schmitt P, Lee KS, Weaver K, Miller O, Cooper M, Jazayeri SD, Damron FH, Mills KHG. Intranasal COVID-19 vaccine induces respiratory memory T cells and protects K18-hACE mice against SARS-CoV-2 infection. NPJ Vaccines. 2023 May 13;8(1):68. doi: 10.1038/s41541-023-00665-3. PMID: 37179389; PMCID: PMC10182552.**
